# Heat Treatments of Metastable β Titanium Alloy Ti-24Nb-4Zr-8Sn Processed by Laser Powder Bed Fusion

**DOI:** 10.3390/ma15113774

**Published:** 2022-05-25

**Authors:** Maxwell Hein, Nelson Filipe Lopes Dias, Sudipta Pramanik, Dominic Stangier, Kay-Peter Hoyer, Wolfgang Tillmann, Mirko Schaper

**Affiliations:** 1 Chair of Materials Science (LWK), Paderborn University, Warburger Str. 100, 33098 Paderborn, Germany; pramanik@lwk.upb.de (S.P.); hoyer@lwk.upb.de (K.-P.H.); schaper@lwk.upb.de (M.S.); 2 DMRC—Direct Manufacturing Research Center, Paderborn University, Mersinweg 3, 33100 Paderborn, Germany; 3 Institute of Materials Engineering (LWT), TU Dortmund University, Leonhard-Euler-Str. 2, 44227 Dortmund, Germany; filipe.dias@tu-dortmund.de (N.F.L.D.); dominic.stangier@tu-dortmund.de (D.S.); wolfgang.tillmann@udo.edu (W.T.)

**Keywords:** laser powder bed fusion, Ti-24Nb-4Zr-8Sn, titanium alloy, biomedical engineering, mechanical properties, microstructure, X-ray diffraction

## Abstract

Titanium alloys, especially β alloys, are favorable as implant materials due to their promising combination of low Young’s modulus, high strength, corrosion resistance, and biocompatibility. In particular, the low Young’s moduli reduce the risk of stress shielding and implant loosening. The processing of Ti-24Nb-4Zr-8Sn through laser powder bed fusion is presented. The specimens were heat-treated, and the microstructure was investigated using X-ray diffraction, scanning electron microscopy, and transmission electron microscopy. The mechanical properties were determined by hardness and tensile tests. The microstructures reveal a mainly β microstructure with α″ formation for high cooling rates and α precipitates after moderate cooling rates or aging. The as-built and α″ phase containing conditions exhibit a hardness around 225 HV5, yield strengths (YS) from 340 to 490 MPa, ultimate tensile strengths (UTS) around 706 MPa, fracture elongations around 20%, and Young’s moduli about 50 GPa. The α precipitates containing conditions reveal a hardness around 297 HV5, YS around 812 MPa, UTS from 871 to 931 MPa, fracture elongations around 12%, and Young’s moduli about 75 GPa. Ti-24Nb-4Zr-8Sn exhibits, depending on the heat treatment, promising properties regarding the material behavior and the opportunity to tailor the mechanical performance as a low modulus, high strength implant material.

## 1. Introduction

Nowadays, there is still a lack of individualized implants, facing safety and risk concerns [1]. Individually adapted implants may reduce revision surgeries and rehabilitation times. They can also restore joint kinetics and improve implant fixation during healing [2]. Patient-specific, customized implants can significantly increase the success rate of the medical treatment of a patient [3,4]. Additive manufacturing (AM) and, specifically, laser powder bed fusion (LPBF) provide the possibility to manufacture these customized implants [5]. LPBF is a powder-based AM technology in which parts are built layer by layer using a laser beam melting the specified area. This layer-wise fabrication of parts leads to geometrical freedom, with few design constraints, and the possibility to manufacture, economically, down to a batch size of one [6,7].

Titanium and titanium alloys are receiving increasing attention in different industries such as aerospace and especially medical industries, due to their superior mechanical performance, as well as their excellent corrosion resistance and biocompatibility [8,9,10]. For most applications and biomedical use cases, (α + β) phase titanium alloys, such as Ti-6Al-4V or Ti-6Al-7Nb, are used with the main disadvantage of a relatively high Young’s modulus around 110 GPa, which is over 3 to 4 times higher compared to the cortical human bone [11]. Titanium alloys are often used for load-bearing implants, where this mismatch between the implant and surrounding bone leads to stress shielding [12]. The difference in stiffness leads to a reduced loading of the bone. Stress shielding is an adaption of bone cells to varying stress states in the bone and, therefore, may result in bone density reduction and implant loosening [13]. Recent studies reflect the development of titanium alloys towards low elastic moduli accompanied by high strengths, and non-toxic and non-allergic elements [14,15,16,17]. Trying to increase the biocompatibility by substituting the toxic elements aluminum and vanadium, and still obtaining sufficient mechanical properties, the β titanium alloy Ti-24Nb-4Zr-8Sn (Ti2448) was developed. The Ti2448 alloy exhibits a low Young’s modulus of 42 GPa to 55 GPa and a high strength of 800 MPa to 1200 MPa, depending on processing and heat treatment routes [18,19,20,21]. The biocompatibility and corrosion behavior are improved through the substitution of the hazardous alloying elements with non-toxic elements such as niobium, zirconium, and tin. Ti2448 is a promising alloy, containing non-toxic, biocompatible elements, but is also compromised in terms of microstructural features and mechanical properties, due to the high concentration of β phase stabilizing elements. Analogous to pure titanium, the α phase has a hexagonal closed packed (hcp) structure at ambient temperature. Above 882 °C, titanium consists of body-centered cubic (bcc) structures, which is the β phase. The β phase becomes stable and can be maintained in the metastable state below 882 °C by adding β stabilizers. The amount of β stabilizer to obtain purely β phases at ambient temperature depends on the molybdenum equivalency Mo_eq_, which is an empirical rule derived from an analysis of binary titanium alloys, and can be described as follows [22,23,24,25,26]:

Mo_eq_ = 1.0 Mo + 0.67 V + 0.44 W + 0.28 Nb + 0.22 Ta + 1.6 Cr + 2.5 Fe...− 1.0 Al [wt. %]. (1)

In general, the addition of 10 wt. % molybdenum suppresses the transformation from β to α upon quenching. Below a Mo_eq_ of 10, the alloy is metastable [22,26]. The effect of molybdenum is, on the one hand, a reduction in the critical cooling rate to retain the β phase and, on the other hand, a significant reduction in the martensitic start temperature M_Sα′_ [25]. Bania’s equation for the Mo_eq_ includes a factor for aluminum. The aluminum equivalent Al_eq_ is added to reflect the tendency to support the formation of the α phase concerning the α stabilizers aluminum, tin, oxygen, and nitrogen as follows [26,27]:

Al_eq_ = 1.0 Al + 0.17 Zr + 0.33 Sn + 10 O + 10 N [wt. %]. (2)

In the condition of energy disturbance, such as heat treatment and deformation, the metastable bcc β phase decomposes into the hcp α phase, the hcp α′ martensite phase, the orthorhombic α″ martensite, or/and the ω phase [27,28,29,30,31,32]. The α′ martensite formation in titanium alloys, in general, results in increasing tensile strength and hardness accompanied by decreasing plasticity [33,34]. The formation of α″ martensite under rapid quenching tends to reduce hardness, tensile, and fatigue strength [18,35,36,37,38]. Additionally, the ω phase is strengthening the titanium alloy in a certain volume fraction range, but also can lead to embrittlement [39,40]. Heat treatments of metastable β titanium alloys show promising features to improve and enhance the mechanical properties and are considered for different fields of applications, including aerospace or medical applications [22,26,41,42].

Various titanium alloys have already been manufactured by LPBF, such as Ti-6Al-4V [43,44,45] and Ti-6Al-7Nb [46,47,48,49,50]. As described, these alloys obtain high elastic moduli compared to human bones, which could lead to stress shielding, and contain, in part, hazardous elements such as aluminum and vanadium, which are referred to allergic reactions, neurotoxic effects, and probably Alzheimer’s disease [51,52,53]. Therefore, in this study, the low-modulus metastable β titanium alloy Ti2448 is manufactured by LPBF and investigated. The complex phase transformation in combination with the LPBF is unknown and these investigations are to contribute to understanding the mechanisms. Subsequent heat treatments are applied to modify and adjust the mechanical properties. To tailor the material behavior and to achieve sufficient mechanical properties, different heat treatments are conducted. Conclusively, the effects of different microstructures in the as-built and heat-treated conditions on the mechanical behavior is determined.

## 2. Materials and Methods

**Figure 1:**
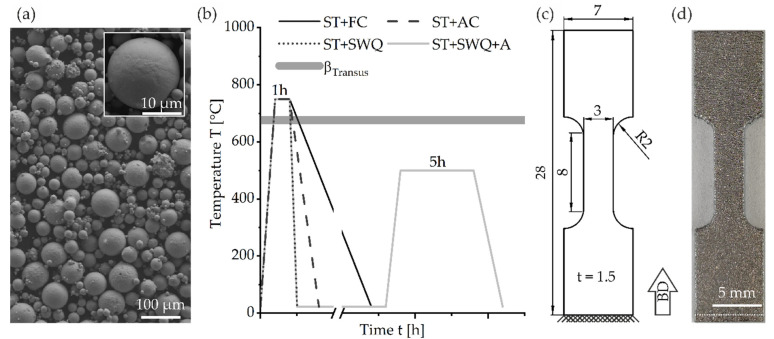
(**a**) SEM image of the morphology of initial Ti2448 powder, inset showing a spherical powder particle in higher magnification; (**b**) schematic overview of the heat treatments (ST = solution treatment, FC = furnace cooling, AC = air cooling, SWQ = slow water quenching in a glass ampule, A = aging) as well as the β_Transus_ temperature; (**c**) geometry in reference to the building direction (BD) of the tensile specimens; (**d**) image of an as-built sample, support structure below the white dashed line.

The Ti2448 powder was gas atomized by GfE Metalle und Materialien GmbH (Nürnberg, Germany) and examined concerning particle size distribution with a Mastersizer 2000 (Malvern Panalytical GmbH, Kassel, Germany) using laser diffraction. The powder is mainly spherical, see Figure 1a. It has a nominal particle size distribution between 28.5 µm (D_10_) and 73.9 µm (D_90_) with a log-transformed normal distribution centered at 45.9 µm (D_50_). The chemical composition of the initial Ti2448 powder was determined by the Revierlabor (Chemische Laboratorien für Industrie und Umwelt GmbH, Essen, Germany) by X-ray fluorescence analysis, combustion analysis with infrared detection, and standard carrier gas hot extraction. The measured composition is compared to the target values and the LPBF manufactured specimens. The chemical composition of the as-built specimens was determined using the Bruker Q4 Tasman optical emission spectrometer (OES, Bruker AXS GmbH, Karlsruhe, Germany).

The specimens were manufactured using an LT12 SLM machine (DMG MORI AG, Bielefeld, Germany) in an inert argon atmosphere containing less than 1000 ppm oxygen to minimize oxidation of the molten pool and the risk of introducing contaminants. The machine is equipped with a 400 W fiber laser with a spot size of 35 µm. Specimens were manufactured using a laser power P = 225 W, with a laser scanning speed v = 1.5 m s^−1^ and hatch distance h = 0.1 mm to obtain a relative density of more than 99.5%. As the contour parameters, a laser power P_c_ = 150 W and a scanning speed v_c_ = 0.4 m s^−1^ were applied. The layer thickness was kept constant at 50 µm, while the layer-wise rotation of the scanning vectors of 67°, and 5 mm stripes were applied. The building platform was preheated to 200 °C.

The as-built specimens were studied and compared to heat-treated specimens to analyze the differences in microstructure and mechanical properties. For identification of the effects of heat treatments, four different treatments were conducted in evacuated glass ampules in a Nabertherm furnace N41/13 (Nabertherm GmbH, Lilienthal, Germany). Solution treatment (ST) was performed at 750 °C for 1 h for all heat treatments followed by different cooling rates. Furnace cooling (FC), air cooling (AC), and slow water quenching (SWQ) of the glass ampules in water were applied. Additional specimens were aged at 500 °C for 4 h after SWQ (SWQ+A) with subsequent AC, see Figure 1b [54].

The crystalline phases were analyzed by X-ray diffraction (XRD) using a D8 Advance diffractometer (Bruker AXS GmbH, Karlsruhe, Germany) equipped with a polycapillary parallel X-ray lens of 2 mm and a LynxEye silicon strip detector (Bruker AXS GmbH, Karlsruhe, Germany). The specimens were ground with sandpaper (grain size 2500) and then vibration polished for 24 h on a VibroMet (Buehler, ITW Test & Measurement GmbH, Düsseldorf, Germany). The XRD measurements were performed with Cu-Kα1 radiation (λ = 1.5406 Å) at an acceleration voltage of 40 kV and a current of 40 mA. The diffractograms were obtained in Bragg-Brentano geometry over a scanning range of 2θ within 30° and 90° with a scan step of Δ2θ = 0.034° and an exposure time of 1 s. In addition, the lattice parameters of the hcp α, orthorhombic α″, and bcc β phases were determined.

For microstructural and hardness investigations, the specimens were ground and vibration polished. KOH-solution (32% H_2_O; 8% H_2_O_2_; 60% KOH (40%)) was used for etching the specimens from 2.5 min to 5 min. The etched specimens were investigated with a light microscope (LiMi) Zeiss Axiophot (Carl Zeiss AG, Oberkochen, Germany). Powder morphology and microstructure were examined with a scanning electron microscope (SEM) Zeiss Ultra Plus (Carl Zeiss AG, Oberkochen, Germany).

Further examination of the microstructure was performed using transmission electron microscopy (TEM). Thin foils were prepared. Slices of ≈400 μm thickness were cut employing the Struers Sectom-5 (Struers GmbH, Willich, Germany). The slices were ground using SiC abrasive papers to a thickness of ≈100 μm. Then, 3 mm diameter discs were punched out of the slices. The discs were further thinned with Struers Tenupol-5 (Struers GmbH, Willich, Germany) using an electrolyte containing 5% perchloric acid-methanol solution at a voltage of 21 V, a current of 16 mA, and at a solution temperature of −23 °C. TEM was performed using a JEOL JEM-ARM 200F (JOEL Ltd., Tokyo, Japan). TEM, high-resolution TEM (HRTEM), high angle annular dark-field scanning TEM (HAADF-STEM), and energy dispersive spectroscopy (EDS) was performed. EDS maps were measured on selected regions of the HAADF-STEM images with a 5 nm step size and 2 s dwell time per step.

The Vickers hardness was measured on surfaces perpendicular to the build direction with a hardness tester KB 30 FA (KB Prüftechnik GmbH, Hochdorf-Assenheim, Germany) according to Vickers HV5. A minimum of two specimens per condition were tested with 30 hardness indentions across the surface of each specimen.

The monotonic tensile tests were performed utilizing a servo-hydraulic test-rig MTS 858 Tabletop System (MTS Systems Corporation, Eden Prairie, MN, USA) equipped with a 20 kN load cell and an extensometer 632.29F-30 (MTS Systems Corporation, Eden Prairie, MN, USA). The design of the miniature dogbone specimens is according to DIN EN ISO 6892-1, but does not fulfill the proportional specimen criterium [55]. The loading direction was parallel to the building direction (BD). The geometry and the BD are depicted in Figure 1c. An as-built sample is shown in Figure 1d. The tensile test procedure corresponded to a displacement-controlled execution with a crosshead speed of 1.5 mm min^−1^ according to DIN EN ISO 6892-1 [55].

## 3. Results and Discussion

### 3.1. Chemical Composition

The chemical composition of the initial Ti2448 powder was determined and compared to the target values and the LPBF manufactured specimens, see Table 1. The initial powder niobium content is slightly higher than prescribed, whereas the tin content is slightly lower. Due to the different melting temperatures of the alloying elements, it is challenging to obtain the desired chemical configuration of the initial powder. The contents of zirconium and tin in the as-built specimens are in the prescribed range, the content of niobium is above 9 wt. %. The chemical composition could be affected by the vaporization of the alloying elements during the fabrication process [56,57,58,59,60]. Nevertheless, the oxygen content could be increased due to residual oxygen in the process chamber and the oxygen affinity of titanium at elevated temperatures [61,62]. Based on Equations (1) and (2), the Mo_eq_ is calculated for the initial powder state to be 2.94. Based on the chemical composition of the alloy, one may assume that the alloy is a metastable β to β rich titanium alloy [22,26].

### 3.2. Phase Constituents and Microstructure

**Figure 2:**
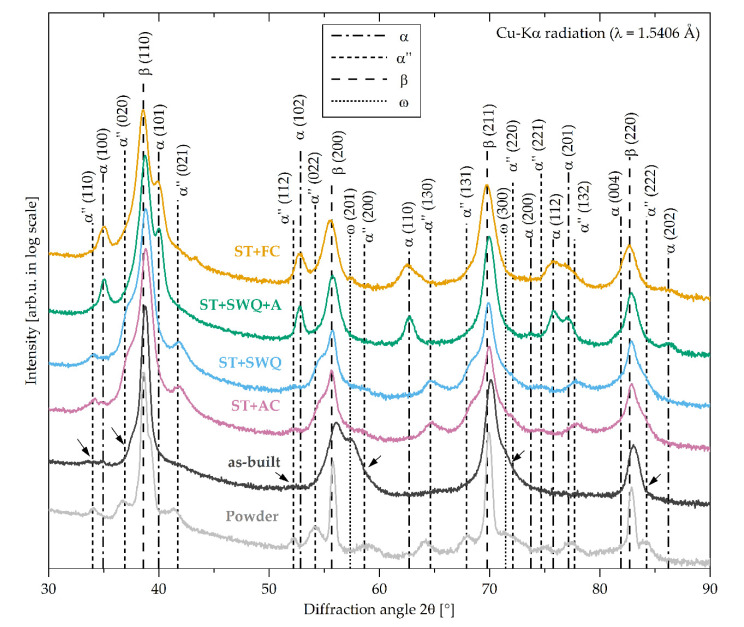
X-ray diffractograms of Ti2448 in as-built, ST+FC, ST+AC, ST+SWQ, and ST+SWQ+A conditions as well as the initial powder; black arrows indicating overlay of α″ phase peaks in the as-built condition; not depicted in the diagram: α″ orthorhombic (111) (superposition with β (110)), α″ orthorhombic (113) (superposition with β (211)); α hexagonal (002) (superposition with β (110)), and α hexagonal (103) (superposition with β (211)).

The microstructure of Ti2448 highly depends on the processing route and the heat treatment. The XRD diffractograms of the LPBF processed Ti2448 in the initial powder, as-built, and heat-treated conditions are shown in Figure 2. It can be observed that Ti2448 mainly consists of the cubic β phase (space group *Im*$\bar{3}$*m*) and, depending on the condition, also contains low amounts of the hexagonal α phase (space group *P*6_3_/*mmc*), orthorhombic α″ martensite (space group *Cmcm*), or presumably hexagonal ω phase (space group *P*6/*mmm*). Compared to the β phase, the Bragg reflections of the remaining phases are broader, indicating a high degree of microstrain (deformed crystal lattice) and/or small grain sizes [63,64]. The Ti2248 powder contains the α″ phase next to the β phase. For Ti2248 in the as-built state, the Bragg reflections of the β (200) and β (211) planes show a shoulder formation towards higher angles, indicating a superposition with another Bragg reflection. At these angles, the Bragg reflections may originate from the ω phase. A clear assignment is not possible due to the overlapping and broad reflections. Contrarily, the heat-treated Ti2248 alloys possess either the α″ or α phase. The microstructure and subsequent transformation behavior are highly sensitive to the chemical composition and various cooling rates from the β phase field. In general, metastable β decomposes in the α (hcp), the α′ martensite (hcp), the α″ martensite (orthorhombic), and the ω phases [27,28,29,30]. The transformation can be tailored by employing heat treatments, thereby controlling the content of β stabilizers in the β phase through the cooling conditions [64,65,66]. For niobium concentrations less than 13 wt. % in Ti-Nb alloys, the α′ martensite is usually present [37]. Therefore, the Ti2248 alloy with its higher niobium content is expected to contain the ω or α″ phase inside a β matrix. With decreasing temperature, the instability of the bcc lattice in β phase alloys increases. As described by Moffat and Larbalestier for Ti-Nb alloys, the α″ and ω phases compete to evolve in an unstable lattice [67]. The β phase decomposition is determined by the quench rate, while the two decomposition modes to α″ or ω phase are mutually exclusive, due to the different transformation variants in metastable titanium alloys, as observed by Duering et al. [68]. For higher cooling rates, the formation of α″ martensite is favorable, while material cooled with lower rates comprise β and ω phase. The lower the cooling rate, the more complete is the collapse of the {222}_β_ planes and the larger the ω precipitates should be [67]. It is assumed that the cooling condition in the LPBF process fosters the formation of the ω phase. As already proposed by Qi et al., a continuous diffusional-displacive β → α″ → α transformation may be present [69]. High cooling rates, such as in SWQ, AC, and during the powder fabrication process, promote the α″ phase formation, while for slow cooling rates such as in FC or for aging treatments, the α″ phase was not detected, see Figure 2. The α″ phase can be regarded as the intermediate stage in between the transformation from bcc to hcp. In the as-built condition, only minor reflections of the α″ phase are observable and, in addition, some of the peaks of other phases are broadened, probably due to the overlay with the α″ phase peaks, see black arrows in Figure 2. It is assumed, that due to insufficient undercooling during the process and/or decomposition through in situ heat treatments, the formation of α″ martensite is inhibited, suppressed, or reversed [34,70,71]. Heat treatments, including slow cooling or aging, enable the decomposition of α″ and the precipitation of the α phase [72]. In summary, slow cooling from the β phase-field, as well as aging, results in the formation of α precipitates. Higher cooling rates result in the martensitic formation of the α″ phase, while in the as-built state, at intermediate cooling rates and/or in-process heat treatment, the formation of the ω and α″ phases seems to be supported, which is mutually competitive.

The lattice parameters of the hcp α phase, orthorhombic α″ martensite, and bcc β phase of Ti2448 in different conditions were determined from the XRD diffractograms and are summarized in Table 2. For the hexagonal ω phase, the calculation of the lattice parameters was not possible due to the overlapping of the Bragg reflections. The lattice parameter of the β phase is in the range between 3.287 Å and 3.303 Å. Deviations can be caused by distortions of the lattices, interference between phases, different chemical compositions, and therefore various atom radii. As calculated by XRD, the α phase exhibits lattice parameters of a = 2.959 Å and c = 4.758 Å for the furnace cooled condition and a = 2.959 Å and c = 4.726 Å for the aged condition. The obtained values for the α and the β phase follow the results of previous studies [62,73]. The α phase lattice parameters of the furnace-cooled determined by TEM are a = 3.191 Å and c = 5.045 Å and deviate from the other values. The orthorhombic α″ phase shows lattice parameters of approximately a = 3.1 Å, b = 4.9 Å, and c = 4.7 Å for the XRD calculation and about a = 3.28 Å, b = 4.88 Å, and c = 4.617 Å for the TEM analysis, which slightly differ from reported values of deformation-induced α″ martensitic transformation [74,75]. It is assumed that the thermal formation of the α″ phase differs from mechanically induced transformations, leading to slightly different lattice parameters.

**Figure 3:**
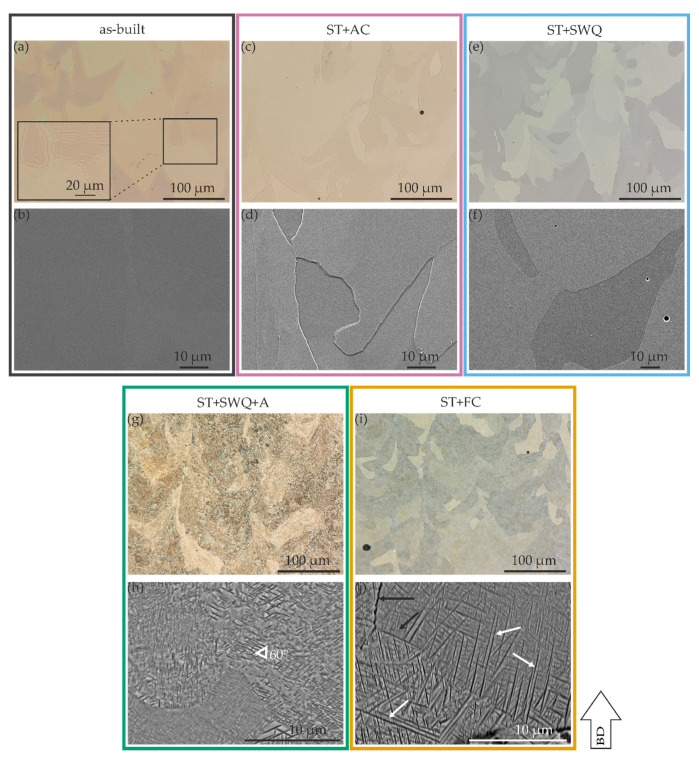
LiMi images of KOH-etched as-built (**a**) inset showing the black box in higher magnification), ST+AC (**c**), ST+SWQ (**e**), ST+SWQ+A (**g**), and ST+FC (**i**) conditions; and SEM images of KOH-etched as-built (**b**), ST+AC (**d**), ST+SWQ (**f**), ST+SWQ+A (**h**), and ST+FC (**j**) conditions; building direction (BD) for all conditions is indicated by the arrow.

The KOH-etched surfaces of the different conditions are depicted in Figure 3. Figure 3a,b demonstrate the as-built condition. β grain boundaries are observable in the LiMi and SEM images. Laths are visible in the grains and in the inset in (a), which is presumably referred to as the α″ martensitic phase. The ω phase was not detected. The air-cooled and water quenched conditions have a similar microstructure, see Figure 3c–f, respectively. Coarse grains are observed for both conditions. In contrast to the as-built condition, no α″ phase laths are observable in the microscopic images for the AC and SWQ condition, although the XRD measurements suggest this. Figure 3g,h show the LiMi and SEM images of the aged specimen. The prior β grains are not as clearly visible as in the other conditions. A very fine, diffuse microstructure is visible in the LiMi, which turned out to be α precipitates at a higher magnification in the SEM. Primary β grain boundaries are visible in the SEM image of the aged condition, see Figure 3h. The initial microstructure, such as in the as-built condition, is visible in the furnace cooled specimen, see Figure 3i. Yet, the SEM close-up in Figure 3j indicates α precipitates similar to those in the aged specimens, but not as homogeneous distributed. As observed by Ren et al., aging has a significant effect on the precipitation behavior of the secondary α phase in titanium alloys [76]. The ST+FC specimens exhibit an irregular distribution and heterogeneous size of intra-granular α precipitates (white arrows) with grain boundary α (black arrows), see Figure 3j. An adapted heat treatment (ST+SWQ+A) can modify the microstructure to achieve a uniform size and orientation (approximately 60°, white triangle) of the acicular α precipitations, see Figure 3h. Varying the aging time and temperature can tailor the morphology of the intra-granular α precipitates and the grain boundary α phase can be coarsened and, therefore, adjust the mechanical behavior [76,77].

A uniform microstructure with grain sizes less than 50 nm was observed by Hao et al. and Li et al. in cold-rolled sheets of Ti2448 [18,20]. The microstructure of hot-forged and cold-rolled Ti2448 was investigated and compared by Li et al. [19]. The hot-forged material consists of equiaxed β grains with a grain size of around 5 µm, divided into subgrains with sizes less than 1 µm. Aging of the hot-forged alloy resulted in α phase precipitations with a needle-like shape. The cold-rolled Ti2448 has a β + α″ microstructure with coarse grains but also nanostructured regions. Yang et al. cold-rolled previous hot-forged Ti2448 cylinder and performed subsequent solution treatment at 900 °C with water quenching (ST) or flash treatment at 700 °C with air cooling (FT). The resulting microstructures were homogenous single β phase with grain sizes about 50 µm and 7 µm, respectively [78]. The microstructure of Ti2448 processed by warm swaging and warm rolling is presented by Hao et al. [79]. The hot-forged alloy showed an equiaxed β phase microstructure with grain sizes of 100 µm, consisting of equiaxed subgrains with sizes of around 1 µm. After warm swaging, the microstructure became swirled marble-like, with differences from the surface to the core. Subsequent warm rolling resulted in a homogeneous microstructure comprising β phase with grain sizes less than 200 nm and nanosized α phase. Zhang et al. observed a microstructure for as-hot-rolled Ti2448 with grains around tens of microns, containing subgrains of the sizes of hundreds of nanometers, and consisting of single β phase without the formation of ω phase or α″ martensite [21].

**Figure 4:**
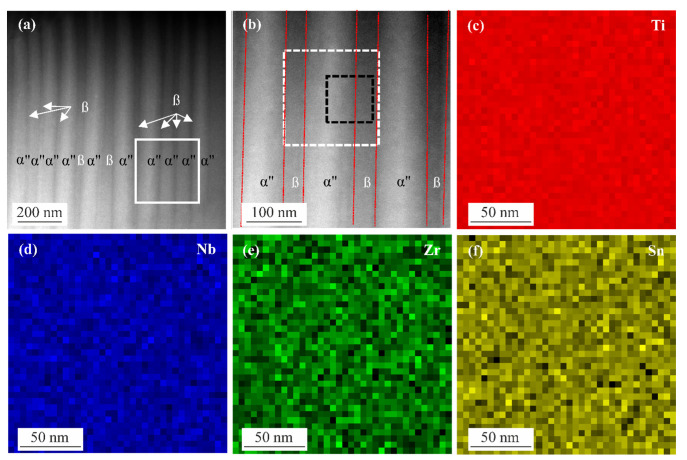
(**a**) HAADF STEM image of the as-built specimen, (**b**) HAADF STEM image of the white square region in (**a**), brighter laths are α″ martensite phase, while the surrounding area is the β matrix, red, dashed lines demarcate the boundary between α″ martensite and β phase; EDS maps from the white, dashed square in (**b**) and chemical distribution of (**c**) titanium, (**d**) niobium, (**e**) zirconium, and (**f**) tin.

Figure 4 shows the HAADF-STEM images and EDS maps of the as-built specimen. Figure 4a,b illustrate the nanostructure consisting of alternately α″ martensite and β titanium laths. Figure 4b is the magnification of the white square region in (a). The images prove the presence of parallel plates. The thickness of the plates varies between 42 and 85 nm. The plates are orthorhombic α″ martensite phase (bright laths) and the surrounding matrix is β phase (dark laths). The EDS maps for titanium, niobium, zirconium, and tin are depicted in Figure 4c–f and are taken from the white, dashed square in (b). The composition of the alloying elements is uniform in the α″ plates and β matrix. This indicates the diffusion-free nature of the orthorhombic α″ martensite phase formation.

**Figure 5:**
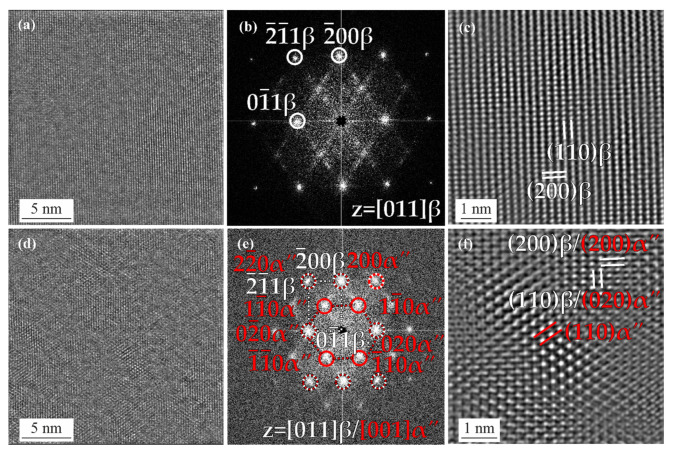
HRTEM, FFT, and Fourier filtered HRTEM images from the black, dashed square region in Figure 4b; (**a**) HRTEM and (**b**) FFT images of the β phase in (**a**), zone axis is [011]β; (**c**) higher magnification Fourier filtered HRTEM image of the β matrix in (**a**); (**d**) HRTEM and (**e**) FFT images of the α″ and β phase in Figure 4b, zone axis in is [011]β/[001]α″; (**f**) higher magnification Fourier filtered HRTEM image of the α″ and β phase in (**d**).

HRTEM, FFT, and Fourier filtered HRTEM images of the as-built specimen from the black, dashed square region in Figure 4b are shown in Figure 5. Figure 5a–c are from the β phase, whereas Figure 5d–f are from the α″/β interface region. The HRTEM image in Figure 5a highlights the lattice of the β phase. The corresponding FFT image in (b) is captured from the [011]β zone axis and shows the 0$\bar{1}$1β, $\bar{2}$00β and $\bar{2}\bar{1}$1β spots. Figure 5c is the zoomed-in image of (a), indicating the (110)β and (200)β planes. Figure 5d shows the HRTEM image from the lattice fringes of the orthorhombic α″ martensite phase and β phase lattice planes. Figure 5e is taken from the [011]β and [001]α″ zone axis. The FFT image in (e) presents the 0$\bar{1}$1β, $\bar{2}$00β, and $\bar{2}\bar{1}$1β spots as well as the 0$\bar{2}$0α″, 200α″, 2$\bar{2}$0α″, and $1\bar{1}$0α″ spots. Multiple spots are observed to overlap with each other: (i) 0$\bar{2}$0α″ and 0$\bar{1}$1β, (ii) 200α″ and $\bar{2}$00β and (iii) 2$\bar{2}$0α″ and $\bar{2}\bar{1}$1β. From the FFT image, the orientation relationship between the β and α″ martensite phase is [001]α″∥[011]β, [0$\bar{2}$0]α″∥[0$\bar{1}$1]β, [2$\bar{2}$0]α″∥[$\bar{2}\bar{1}$1]β and [200]α″∥[$\bar{2}$00]β. Figure 5f illustrates a magnified HRTEM marking the fringes from the (200)β, (200)α″, (110)β, (020)α″, and (110)α″ lattice planes.

**Figure 6:**
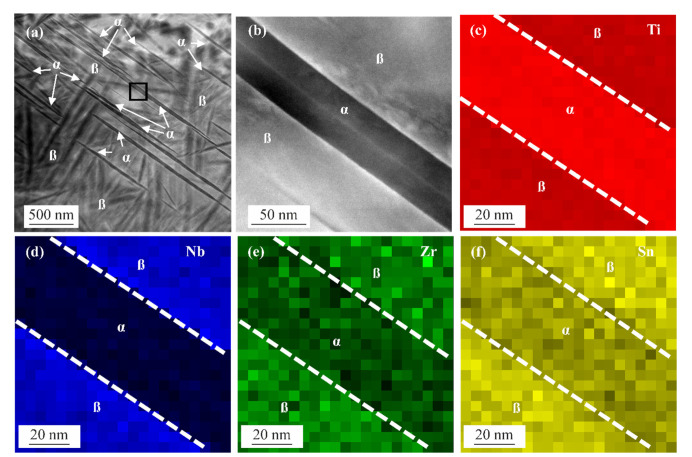
(**a**) HAADF STEM image of the ST+FC specimen with α phase laths inside the β phase matrix; (**b**) HAADF STEM image of the black square in (**a**), showing a higher magnification of an α phase lath surrounded by the β phase; EDS maps and chemical distribution of (**c**) titanium, (**d**) niobium, (**e**) zirconium, and (**f**) tin, demonstrating titanium enrichment inside the α phase (higher color intensity) and titanium depletion inside β phase, and enrichment of niobium, zirconium, and tin inside the β phase (higher color intensity) and depletion inside α phase, respectively.

HAADF-STEM images of the ST+FC specimens are depicted in Figure 6a,b, showing the α and β phases. The α phase is present in the form of plates (white arrows) inside the β matrix. The EDS maps in Figure 6c–f are taken from the region in (b), showing the distribution of titanium, niobium, zirconium, and tin in the α and β phases. A depletion (lower color intensity) of niobium and an enrichment (higher color intensity) of titanium is observed in the α phase, indicating a diffusional mechanism of α phase formation due to the diffusion of mainly niobium. Zirconium and tin are stabilizing or neutral to the β phase-formation of titanium [80,81]. Minor differences in the distribution of zirconium and tin in terms of depletion can be observed between the α and β phases.

**Figure 7:**
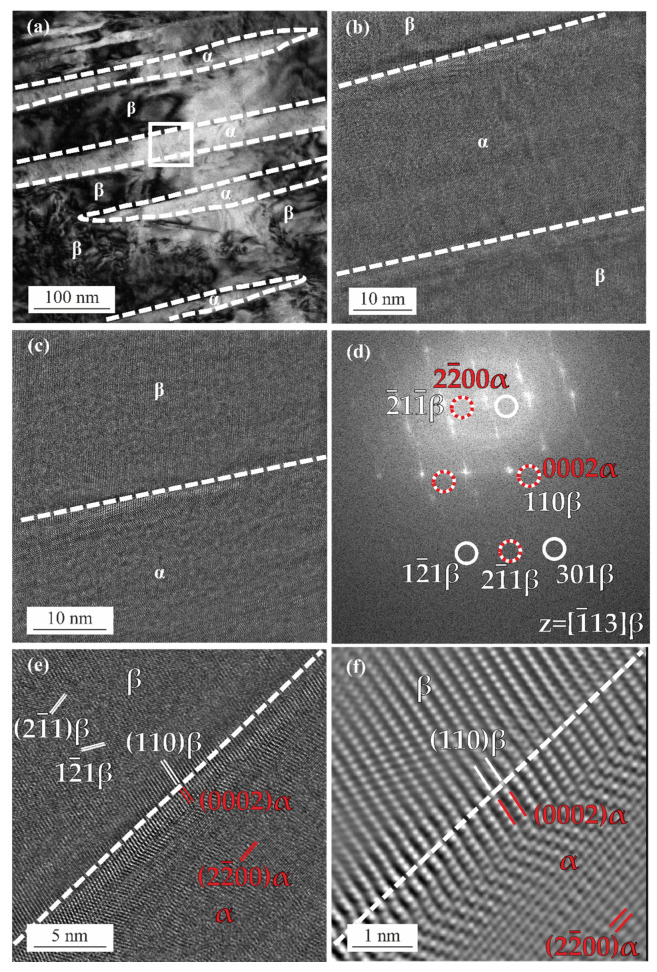
(**a**) TEM image of α phase and β phase in the ST+FC specimen; (**b**) HRTEM image of the α lath and β phase; (**c**) HRTEM image of the upper α/β interface in (**b**); (**d**) FFT image of the area in (**c**) with [113]β zone axis; (**e**) magnified HRTEM image of the α/β interface in (**c**) with the (2$\bar{1}$1)β, (1$\bar{2}$1)β, (110)β, (0002)α, and (2$\bar{2}$00)α planes marked; (**f**) Fourier filtered HRTEM image showing the α/β interface in (**e**) with the (110)β, (0002)α, and (2$\bar{2}$00)α planes marked.

The TEM image of the ST+FC specimen depicted in Figure 7a shows the α phase plates and the β phase matrix. Figure 7b illustrates the HRTEM image of the α plate taken from the white square region in (a). Figure 7c is a magnified view of the top β/α interface in (b). The FFT image in Figure 7d is calculated from (c) with the zone axis of [$\bar{1}$13]β. The 0002α spot overlaps with the 110β spot, indicating that (0002)α∥(110)β. Figure 7e is a close-up HRTEM image of the β/α interface of (c), marking the fringes from the (0002)α, (110)β, (2$\bar{2}$00)α, (1$\bar{2}$1)β, and (2$\bar{1}$1)β lattice planes. At the β/α interface, the lattice fringe of (0002)α is parallel to (110)β. A magnified Fourier filtered image of the β/α interface is presented in Figure 7f, illustrating the (0002)α∥(110)β relationship.

Metastable β titanium alloys tend to decompose to the ω, α, and α″ phases. The ω phase probably occurs at lower temperatures, as described by Ohmori et al. [82]. In the as-built condition, the ω phase was not observed during TEM investigations, but, due to the XRD results, is assumed to be present in this condition. For the furnace cooled conditions, nevertheless, the α phase laths probably nucleate at the ω/β interfaces and grow in the β matrix, consuming the ω phase particles, as described by Ohmori et al. during quenching from the β phase region. The α″ martensite plates nucleated preferentially at the β grain boundaries [82].

### 3.3. Mechanical Properties

**Figure 8:**
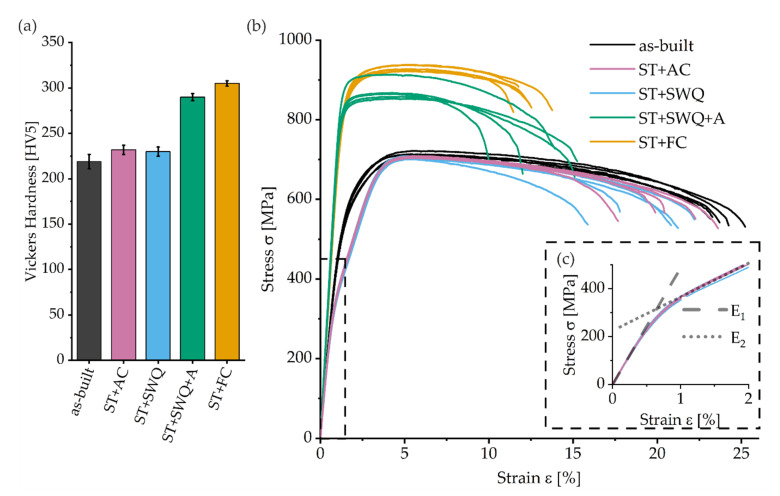
(**a**) Vickers hardness for different conditions; (**b**) stress–strain curves of different conditions; (**c**) inset of the dashed boxed in (**b**) presenting the nonlinear elastic behavior of the ST+AC and ST+SWQ conditions.

The hardness of the additively manufactured Ti2448 in as-built and heat-treated conditions is summarized in Figure 8a. In the as-built condition, the hardness is 219 ± 8 HV5 and slightly lower but in good agreement with other research on LPBF-fabricated Ti2448 with near full dense (230 HV0.5 [83], 240 HV1 [54], 230 HV0.5 [84]), and lower than for EBM-fabricated Ti2448 (255 HV [85]). For conventional conditions, the hardness varies between 215 HV (hot-forged), 230 HV (warm swaged), and 265 HV (warm rolled) [79]. For ST+AC and ST+SWQ conditions, the hardness is slightly higher, 232 ± 5 HV5 and 230 ± 5 HV5, respectively, compared to the as-built condition, probably due to the presence of α″ martensite [18,35,36,37,38]. The ST+SWQ+A heat treatment leads to a hardness of 290 ± 4 HV5. The highest hardness results in ST+FC condition with 305 ± 3 HV5. The increased hardness of the aged and furnace cooled condition is assumed to be precipitation hardening due to α precipitates formation in the β matrix [69,86]. The hardness values are summarized in Table 3.

The stress–strain curves of the as-built and heat-treated specimens of Ti2448 are depicted in Figure 8b. The lowest ultimate tensile strength (UTS) of 700 ± 6 MPa and the highest fracture elongation A of 22 ± 1% were measured for the as-built condition. AC or SWQ after ST result in similar UTS of approximately 705 MPa and elongations of around 20%. The Young’s moduli E_1_ of the as-built, ST+AC, and ST+SWQ conditions are similar at the beginning of the elastic deformation, until a strain of 0.5%. The tensile tests of the ST+AC and ST+SWQ conditions show significant and continuous elastic softening with increasing strain and, therefore, stress. The slope changes for strain values higher than 1% to E_2_ of approximately 14 ± 0.11 GPa, see Figure 8c. The α″ martensite phase is present in these two conditions. Kolli et al. propose deformation mechanisms by conventional slip and stress-induced transformation due to α′ martensite, α″ martensite, ω phase, and deformation twinning, whereas the mechanisms depend on the β phase stability of the titanium alloy [42,87]. With increasing Mo_eq_, the stress-induced deformation mechanisms follow the sequence α′ → α″ → ω + twinning → twinning → twinning + slip → slip. With a Mo_eq_ of around 2.94 and a niobium content higher than 13 wt. %, the alloys are expected to contain the α″ phase and the deformation mechanism is likely to be based on α″ formation [37]. The presence of metastable α″ martensite or ω phase can promote stress-induced transformation and serve as nucleation sites during deformation [42,87]. As described by Furuta et al., both conditions show pseudo-elastic deformation. This behavior is based on stress-induced α″ martensite, which is determined through in situ XRD measurements during tensile tests. Along with increasing tensile strain and stress, the α″ phase retransforms to β and can be explained by reversible martensitic transformation, also known as “pseudo-elastic deformation″ [88]. Hao et al. assume stress-induced phase transformation and/or incipient kink bands as the origin of the peculiar elastic behavior [89]. Although the LPFB process leads to α″ martensite microstructure, shown by the TEM investigations, this exceptional pseudo-elastic deformation behavior was not detectable for the as-built condition. The thermal history of each layer during the process may result in a characteristic microstructure of the as-built specimen and, therefore, a unique material behavior. The material undergoes a series of thermal cycles, where the previously fabricated material can be partially re-melted and re-solidified [90,91]. Non-linear elastic behavior of the as-built condition may be attributed to the microstructure, probably characterized by localized distorted region with the elastic strain being located hierarchically in the alloy, as assumed by Furuta et al. [88]. Subsequent aging or FC after ST led to higher strengths, 871 ± 22 MPa and 931 ± 7 MPa, a significant reduction of elongations, 12 ± 2% and 11 ± 1 MPa, and increased Young’s moduli E_1_, 76 ± 3 GPa and 73 ± 1 GPa, respectively. These treatments produce fine dispersion of α precipitates as the principal strengthening mechanism. The aging temperature and time have a significant effect on the size and spacing of the α phase formed and, therefore, on the mechanical performance [92].

The mechanical properties are summarized in Table 3. Rapid cooling during fabrication or heat treatment results in lower strength properties. High cooling rates from above the β_Transus_ temperature result in the formation of the α″ martensite and, consequently, lower strength and elongation, compared to the as-built condition. Previous studies have shown that the α″ martensite phase reduces hardness, tensile, and fatigue properties [36,93]. Through slow cooling or aging treatments at temperatures below β_Transus_, α phase laths can evolve and grow. Annealing treatments and slower cooling lead to strengthening due to enhanced nucleation of the α precipitates [20,94]. Compared to other research on additively manufactured Ti2448, the properties are in good agreement regarding the hardness and UTS, whereas the YS and fracture elongation slightly differ, due to varying proportionality factors concerning the specimen geometry [83]. In comparison to other processing routes, the different heat treatments, in particular the aging and FC, enhance the mechanical properties in terms of UTS and hardness. The properties of the warm swaging and rolling treatments are caused by the ultrafine-grained microstructures, not achievable by the applied treatments [79]. Further work shows similar properties, depending on the processing routes and heat treatment strategy [95], although, for the additively manufactured Ti2448, different treatments show huge potential in improving the mechanical behavior and properties, especially aging or FC. Besides improved UTS and YS remaining similar elongation compared to the conventional process routes, the Young’s moduli increase for these conditions due to the presence of α precipitations. The hardness of conventional processed Ti2448 can be outperformed by the additively processed and heat-treated conditions [19]. Nevertheless, additively manufactured and heat-treated (FC or SWQ+A) Ti2448 achieves similar or better mechanical performances than conventional processed Ti2448 regarding UTS and YS, with slightly increased Young’s moduli and still retained good ductility.

The possibility of in situ α″ martensitic transformations during deformation of the β phase for air-cooled and slow water quenched specimens, during hardness or tensile tests, could be present. The determination is challenging and would require the observation of the specimens at the nanoscopic level during the tests. Further investigations on the stress-induced deformation and formation of the α″ phase are necessary for a comprehensive understanding of the mechanical behavior, exclusively concerning the fatigue behavior and the utilization as an implant alloy. In addition, the individual tailoring of the mechanical properties by adapting the aging heat treatment parameters has to be considered for further research.

## 4. Conclusions

Micro- and nanostructure, as well as Vickers hardness and monotonic tensile properties of Ti2448 manufactured with LPBF, have been studied in the as-built state and heat-treated conditions. Solution heat treatment at 750 °C with various cooling rates (AC, FC, and SWQ) or ST with subsequent aging at 500 °C were performed and compared to the as-built additively manufactured condition. The effect of different heat treatments is determined as follows:

- The as-built condition shows mainly β phase containing acicular α″ martensite phase, as detected via TEM. Air-cooled and water-quenched states exhibit similar microstructures consisting of β grains, investigated by LiMi and SEM. The aged condition exhibits a diffuse microstructure with homogeneous and uniform α precipitates inside the β matrix. The furnace-cooled specimens have a microstructure comparable to the aged conditions but have heterogeneously distributed and coarser α precipitates within the β phase.
- The nanostructure, determined by TEM, of the as-built condition is compared to the furnace cooled condition, which exhibits the best tensile properties regarding hardness, UTS, and YS. The α″ martensite laths were detected inside the β matrix. Based on FFT images, the orientation relationship of the α″ martensite and β phase is determined. The homogeneous distribution of alloying elements in the EDS maps indicates a diffusion-free phase transformation during cooling. The furnace-cooled specimens consist of α precipitates inside a β phase matrix. Based on crystallographic relation, the precipitates are oriented approximately 60° to each other.
- X-ray diffractograms are sensitive to the various heat treatments and, in particular, the cooling rates. High cooling rates, e.g., AC, SWQ, and the powder fabrication process, lead to the formation of the martensitic α″ phase. Low cooling rates (FC) or aging after ST result in the formation of the α phase.
- FC or aging after solution treatment results in a microstructure containing acicular α precipitates in the β matrix, leading to high tensile strength with relatively low ductility. Phase transformation, such as stress-induced α″ phase transformation, probably leads to pseudo-elastic deformation behavior in the air-cooled and slow water-quenched conditions. As the α″ phase was not detected with XRD in the furnace cooled and aged conditions, a linear stress–strain relationship was observable in the elastic range. The as-built conditions show elastic anomaly, which is attributed to the LPBF resulting microstructure. The furnace cooled condition exhibits the best mechanical properties regarding UTS, YS, and hardness with a slightly worse fracture elongation compared to the aged conditions.

Controlling the development and refinement of precipitated α phase in metastable β titanium alloys allows the achievement of an excellent combination of strength and ductility, including superb biocompatible properties. In this work, the furnace-cooled specimens obtain the best mechanical properties regarding hardness, UTS, and YS. Nevertheless, the aged condition has to be considered for further investigations regarding the possibility of tailoring the material properties. Adapted heat treatments enable adjusting the microstructural features in terms of the α precipitates. Furthermore, in situ investigations at the nanoscopic level should be performed to determine and understand the material behavior and possible α″ phase transformation behavior during the tensile tests of the air-cooled and water-quenched conditions. If fully understood, the two-part Young’s modulus can be utilized. Regarding the overall performance, Ti2448 shows promising features regarding the tailoring of mechanical properties and applying an implant material with a low Young’s modulus, as well as adequate and adaptable strength properties.

## Figures and Tables

**Table 1:** Chemical composition of initial powder and LPBF manufactured Ti2448 alloy in wt. %.

Condition	Ti	Nb	Zr	Sn	O	Mo_eq_
Target Value	Bal.	23.5–24.5	3.5–4.5	7.5–8.5	<0.12	2.32
Initial Powder	Bal.	25.2	4.2	7.1	0.11	2.94
as-built	Bal.	>9.0	3.5	8.2	0.37	-

**Table 2:** Lattice parameters, determined by XRD, for hcp α, orthorhombic α″, and bcc β of Ti2448 in the as-built, ST+FC, ST+AC, ST+SWQ, and ST+SWQ+A condition as well as the initial powder; lattice parameters determined by TEM (marked in brackets).

Condition	Hcp α		Orthorhombic α″			Bcc β
	a = b [Å]	c [Å]	a [Å]	b [Å]	c [Å]	a = b = c [Å]
Initial Powder	-	-	3.107	4.924	4.716	3.298
as-built	-	-	-	-	-	3.287
as-built (TEM)	-	-	3.28	4.88	4.617	3.297
ST+FC	2.959	4.758	-	-	-	3.303
ST+FC (TEM)	3.191	5.045	-	-	-	3.294
ST+AC	-	-	3.110	4.860	4.716	3.294
ST+SWQ	-	-	3.121	4.871	4.679	3.295
ST+SWQ+A	2.959	4.726	-	-	-	3.294

**Table 3:** Comparison of average mechanical properties, including the hardness and monotonic tensile properties, of the as-built and heat-treated Ti2448, as well as literature values including conventional processing methods: yield strength YS, ultimate tensile strength UTS, fracture elongation A, and Young’s Moduli E_1_ and E_2_.

Condition	Hardness [HV5]	YS [MPa]	UTS [MPa]	A [%]	E_1_/E_2_ [GPa]
as-built	219 ± 8	490 ± 16	700 ± 6	22 ± 1	49 ± 1/-
ST+AC	232 ± 5	362 ± 7	707 ± 2	20 ± 2	51 ± 2/14 ± 0.1
ST+SWQ	230 ± 5	339 ± 10	705 ± 3	19 ± 2	50 ± 2/14 ± 0.1
ST+SWQ+A	290 ± 4	819 ± 27	871 ± 22	12 ± 2	76 ± 3/-
ST+FC	305 ± 3	805 ± 11	931 ± 7	11 ± 1	73 ± 1/-
LPBF [83]	220–230	563 ± 38	665 ± 18	14 ± 4	53 ± 1/-
Hot rolled [21]	-	700	830	15	46
Hot-forged [19]	230–370	570	750	13	55
Hot-forged [79]	215	-	800	18	52
Warm swaged [79]	230	-	850	14	55
Warm rolled [79]	265	-	1150	8	56

## Data Availability

The data that support the findings of this study are available from the corresponding author upon reasonable request.
